# Petroleum

**Published:** 1862-01

**Authors:** George Hadley

**Affiliations:** Professor of Chemistry and Pharmacy in the University of Buffalo


BUFFALO

### Medical and Surgical Journal

AND REPORTER.

VOL. I. JANUARY, 1862. NO. 6.

### ORIGINAL COMMUICATIONS.

ART. I.—*Petroleum—By* GEORGE HADLEY, M. D., *Professor of Chemistry and Pharmacy in the University of Buffalo.*

The manufacture of Coal Oil, closely followed by the discovery and opening of immense sources of Petroleum in Western Pennsylvania and in other regions, is introducing a new era in the art of illumination. Already a marvelous improvement has been effected in the comfort of many homes. Tallow candles no longer make the darkness visible; whale oil and sperm are abandoned to the railroads and machine shops; and even the clear brilliancy of wax, and stearine and spermaceti suffers eclipse. "Burning fluid," so popular and convenient, is far inferior in economy, safety and illuminating power, to its new rival; and with the single exception of coal gas, these substances appear likely to supplant in common use, every other material employed for purposes of illumination. Not only their brilliancy, but the great abundance and consequent cheapness of the new burning oils, is fast contributing to this result. This is particularly true of Petroleum. The productiveness of the "Oil Wells" is truly astonishing. From some of them Petroleum has continued to flow spontaneously for many months at the rate of several hundred barrels a day. The total product of those on Oil Creek alone has been estimated at not less than ten thousand barrels, or four hundred thousand gallons a day. And in other localities are enormous reservoirs, which are now pouring forth their treasures, stored up for ages, to give a new fulfillment to the ancient fiat, "Let there be light."

But what is Petroleum, and what is its origin? Asphaltum, Mineral Tar, Petroleum, Rock Oil, Seneca Oil, Naptha, are some of the various names of a class of substances closely allied to each other in chemical composition and physical properties, which are called by the general name of "bituminous" substances. They vary in consistence from brittle solids and thick tarry oils, by all degrees, to the most thin and fluid liquids. Usually they are dark colored, black or brown, and have a strong and peculiar "bituminous" odor. They all burn readily with a smoky flame. To the fixed oils, or fats, they do not bear so close a resemblance as the resins, and the volatile or essential oils. Their solubility and their solvent properties are similar to those of the latter, and they burn with the same strongly. luminous and very smoky flame.

Bituminous matters of every kind are undoubtedly of organic origin. Animals and plants have furnished the material out of which they have been made. Imbedded deep in the rocky foundations of the earth, peculiar conditions of decomposition have given rise to these peculiar products.

No one geological formation has sole claim to them. They do not occur exclusively in the coal burning rocks, but are found in older as well as in more recent formations; and their slight specific differences are due in part at least, to this diversity of origin. The conditions which have given rise to their segregation from bituminous rocks, and to their enormous accumulation in certain localities, I shall not now discuss.

The term Petroleum is applied to the liquid bitumens, and from these the refined Petroleum used for burning is manufactured. The first step in its preparation is to subject the crude oil to the process of distillation.—This is conducted in large retorts of wrought or cast iron, holding from a few hundred to one or two thousand gallons, set in brick-work, and connected with suitable condensers for collecting the volatile products of the distillation.

The oils which come over at different stages of the process, differ from each other in several particulars. First in density. At the beginning of the distillation they are very light, and have a specific gravity as low as 660—water being 1000. Gradually they grow heavier till at the close of the process their specific gravity is as high as water. Secondly in volatility; at first they have a low boiling point—much below that of water—and rapidly give off vapor and waste away at common temperatures. As the distillation proceeds their boiling points gradually rise, till at the last we get liquids which require a heat probably as high as 600 or 800° F. to boil them, and which at ordinary temperature give off no vapor, and are nearly or quite as fixed and permanent as the true fats and oils themselves.

Thirdly, these products differ in consistence. The first distillates are very thin and fluid liquids; the last are quite thick and oily, and on exposure to cold, deposit large quantities of paraffine. The last step in the manufacture consists in agitating with chemicals—acids, alkalies, &c., by which the color is improved and the smell rendered less offensive. As soon after this as it becomes clear, it is ready for use.

The burning oil thus prepared should possess the following qualities: First, it must rise in the lamp wick so rapidly as to furnish a large and strong flame that will not diminish or fall for several hours. Should it fail to do this, and should the flame speedily fall, the wick becomes charred, and raising it again only hastens more rapidly the second time the failure of the flame. Secondly, it must be free from the most volatile products of the distillation; otherwise, heavy combustible vapors are given off from it, and may become the cause of dangerous explosions. The best manufacturers always reject or separate these volatile substances. If a lighted match is held for a few moments just above an oil that contains them, a blue flame flashes over its surface, and the whole is immediately ignited.—If properly made, it is difficult to make the burning oil take fire by holding the match over it, or even by dropping it in, and it is almost as safe from accidents as whale or sperm oil.

From the most volatile products of the distillation of Petroleum, liquids may be separated which are but little more than half as heavy as water, and which boil at a lower temperature than ether. It is a mixture of similar volatile and light liquids obtained from coal, in the manufacture of the "Kerosene Oil," which has been recently recommended as an anæsthetic, under the name of Kerosene. This, like the Petroleum products, is without doubt a mixture of various hydro-carbons. But we have as yet no complete account of its nature and composition, and before introducing it into use, it is every way desirable that it should be subjected to a full and accurate scientific investigation. That some of the volatile liquids obtained from Petroleum would prove to have similar anæsthetic properties is not at all improbable.

Resembling the essential oils in character, the refined Petroleum requires a lamp of strong draught to burn without smoking. The ordinary Kerosine lamps effect this tolerably well. But the flame must not be kept too low, or offensive vapors are generated from the best oil. The fault here is in the lamp, or in the mode of using it. When, however, the oil is properly prepared and the lamp well managed, it will give a strong, steady light, and will continue to burn with little or no falling of the flame for many hours, and without the evolution of any products more noxious or offensive than those from the old sources of illumination.

The economy of the Petroleum and some other subjects connected with its manufacture and use, I may possibly discuss on some future occasion.

