# Errata—January Number—Article on Petroleum

**Published:** 1862-02

**Authors:** 


*Errata—January Number—Article on Petroleum:*

| Page. | Line. |
|---|---|
| 162, | 11 from top, before " the resins," insert "to." |
| 162, | 20 from top, for "burning," read "bearing." |
| 162, | 3 from bottom, for " as high as water," read " 849." |
| 163, | 9 from bottom, for "Kerosene," read " Kerosolene," |
| 164, | 1, from top, for " Kerosine," read " Kerosene." |

Prof. Hadley is in no way responsible for these errors. Editor and Printer must be held accountable for these, and many other similar sins.

